# Integrin Alpha-2 as a Potential Prognostic and Predictive Biomarker for Patients With Lower-Grade Glioma

**DOI:** 10.3389/fonc.2021.738651

**Published:** 2021-10-27

**Authors:** Li Lin, Kai Huang, Zewei Tu, Xingen Zhu, Jingying Li, Kunjian Lei, Min Luo, Peng Wang, Chuandong Gong, Xiaoyan Long, Lei Wu

**Affiliations:** ^1^Department of Neurosurgery, The Second Affiliated Hospital of Nanchang University, Nanchang, China; ^2^Department of Scientific Research, East China Institute of Digital Medical Engineering, Shangrao, China; ^3^Institute of Neuroscience, Nanchang University, Nanchang, China; ^4^Department of Comprehensive Intensive Care Unit, The Second Affiliated Hospital of Nanchang University, Nanchang, China

**Keywords:** immune infiltration, prognostic signature, overall survival (OS), lower grade gliomas, integrin alpha-2 (ITGA2)

## Abstract

Diffuse gliomas are the most common malignant brain tumors with the highest mortality and recurrence rate in adults. Integrin alpha-2 (ITGA2) is involved in a series of biological processes, including cell adhesion, stemness regulation, angiogenesis, and immune/blood cell functions. The role of ITGA2 in lower-grade gliomas (LGGs) is not well defined. Firstly, we downloaded RNA sequencing and relevant clinical information from The Cancer Genome Atlas cohort, the Chinese Glioma Genome Atlas cohort, and related immune cohorts. Next, prognosis analysis, difference analysis, clinical model construction, enrichment analysis, and immune infiltration analysis are performed for this study. These analyses indicated that ITGA2 may have clinical application value and research value in LGG immunotherapy. We also detected the mRNA and protein expression of ITGA2 in three LGG cell lines and normal glial cells using quantitative real-time polymerase chain reaction assay and western blot assay. Our study not only offers a novel target for LGG immunotherapy but also can better comprehend the mechanism of the development and progression of patients with LGG. This study revealed that ITGA2 may be a potential prognostic and predictive biomarker for LGG, which can bring new insights into targeted immunotherapy.

## Introduction

According to the malignant degree of gliomas, the World Health Organization has classified them into grade I to IV, which is judged by a variety of histological features accompanied by genetic changes (1). Diffuse gliomas, which include WHO grade II and III gliomas, are the most common malignant brain tumors with the highest mortality and recurrence rate in adults. It is also known as lower-grade gliomas (LGGs) in studies (2). LGG is a diverse primary and malignant brain tumor that frequently arises in young patients and has an indolent course, with a better survival rate than glioblastoma (3), but it always recurs and progresses into glioblastoma. The poor prognosis for patients with LGG shows the diversity of this malignant glioma; therefore, new treatment strategies are needed to continuously improve the prognosis of LGG patients (4). Although radiotherapy and chemotherapy are used to treat patients with glioma, their effectiveness against glioma is not remarkable (5). Therefore, it is very pivotal to find a new prognostic biomarker to enhance the treatment of glioma and increase the understanding of glioma treatment. Integrin alpha-2 (ITGA2), as an important influencing factor on the tumor immune microenvironment, has the potential to become a molecular marker for targeted therapy and the diagnosis of LGG.

ITGA2 belongs to the integrin family, which is pivotal to sustain the integrity of the cytoskeletal–extracellular matrix linkage among all cell adhesion receptors (6). Some research had shown that ITGA2 is a cell transmembrane receptor that assists the adhesion of other several cells to the extracellular matrix (7). In addition, changes of ITGA2 expression affect the immune microenvironment and immunogenicity of tumors (8). A solid tumor is composed not only of cancer cells but also of immune cells, stromal cells, and more, which all may make a difference to LGG progression in a subtle and dynamic way. ITGA2 reportedly is involved in the occurrence and progress of multiple cancers, including colorectal cancer, hepatocellular carcinoma, and breast cancer (9–11). Experts have studied the ITGA2 ligand blockade that significantly obstructed cell migration in glioblastoma (12), but research in LGG has not yet been carried out. To fill this gap, we suggested that ITGA2 can be a robust prognostic and predictive biomarker for LGG.

We investigated the prognostic significance of ITGA2 by bioinformatic analysis in The Cancer Genome Atlas (TCGA) cohort (*n* = 407); the subgroup of patients with high ITGA2 expression usually induced poor overall survival (OS) times and rates. Similar results were obtained in the Chinese Glioma Genome Atlas (CGGA) cohorts, CGGAseq1 (*n* = 420) and CGGAseq2 (*n* = 171). Using clinical patient information, we performed a correlation analysis with ITGA2 expression and established a clinical nomogram to estimate the OS of patients with LGG. Gene Ontology (GO) analysis was implemented to annotate the function of differentially expressed genes, using ITGA2 expression as a boundary. Analysis of the Kyoto Encyclopedia of Genes and Genomes (KEGG) pathway enrichment was also performed as well as Gene Set Enrichment Analysis (GSEA). A single-sample GSEA (ssGSEA) algorithm investigated the relationship between the enrichment level of the 29 immune-associated gene sets and ITGA2 expression. A study on correlation was applied to explore the relationship between ITGA2 and several known immune checkpoints. Our experiments verified that LGG has a higher protein expression and mRNA expression of ITGA2 than normal glial cells. In brief, through a range of comprehensive analyses, we suggested that ITGA2 can be used as a robust prognostic biomarker for patients with LGG to enhance the outcome of LGG.

## Materials and Methods

### Data Acquisition and Pre-processing

In this study, three independent LGG cohorts—TCGA cohort and CGGA seq1 and CGGA seq2 cohorts—were downloaded and used for analysis. Data of immunotherapeutic cohorts were downloaded from IMvigor210 cohort (a BLCA immunotherapy cohort, *n* = 398) (13) and two melanoma immunotherapy cohorts, including GSE78220 cohort (*n* = 26) and GSE91061 cohort (pre-treatment, *n* = 51) (14). Data of single-cell RNA sequencing analysis was downloaded from GSE84465 cohort. The RNA sequencing and relevant clinical information of TCGA cohort were downloaded from the Genomic Data Commons Data Portal website (https://portal.gdc.cancer.gov/). The mRNA expression and clinical data were obtained from the CGGA website (http://www.cgga.org.cn/), and the CGGAseq1 and CGGAseq2 cohorts obtained were regarded as the two external validation sets. The RNA sequencing and relevant clinical information of IMvigor210 cohort were downloaded from https://www.nature.com/articles/nature25501, and other cohorts were downloaded from GEO database. The inclusion criteria for LGG were as follows: (1) patients with WHO grade II or III glioma, (2) patients diagnosed with glioma with OS of more than 30 days, (3) patients with expression data, and (4) patients with primary glioma. After filtering, we screened for LGGs that could be used for additional analysis from the three independent datasets (TCGA, CGGAseq1, and CGGAseq2), which resulted in 407, 420, and 171 patients with LGG, respectively. The transcripts per kilobase million of the three RNA sequence cohorts were converted from fragments per kilobase per million using a previously published formula (15, 16). In addition, we deleted 50 data from IMvigor210 cohort, which were not available (NA) for PDL1 treatment (*n* = 398). Then, the transcripts per kilobase million values were analyzed in the follow-up work.

### Prognostic Role of ITGA2 and Validation

Patients with LGG were split into subgroups of low and high ITGA2 expression on the basis of the median ITGA2 expression in the TCGA and CGGA cohorts. Receiver operating characteristic (ROC) curves and area under the curve (AUC) values were used to estimate the prognostic predictive ability of ITGA2 expression in the three cohorts. Univariate and multivariate Cox regression analyses were conducted to estimate the independent prognostic value of ITGA2 expression.

### Functional Enrichment Analysis

In the TCGA cohort, the “limma” package, with the standards of |log2 (fold change)| >1 and *P* <.05, was used to identify differentially expressed genes (DEGs) between LGG subgroups of low and high ITGA2 expression (17). Overall, 2,486 DEGs were screened out, and GO biological processes (GO-BP) analysis and KEGG analysis were performed on the basis of selected DEGs using the “clusterProfiler” R package (18). In addition, to identify tumor hallmarks enriched in LGG with higher ITGA2 expression, GSEA was performed by the GSEA software platform (version 4.0.1, https://www.gsea-msigdb.org/gsea/index.jsp) (19).

### Construction and Validation of the Nomogram Predictive Model

Univariate and multivariate Cox regression analyses were conducted to validate the independent prognostic factors among ITGA2 expression and included the following clinical factors: 40 → median age WHO grade (II or III), O(6)-methylguanine-DNA methyltransferase (MGMT) promoter methylation status (methylated or unmethylated), isocitrate dehydrogenase (IDH) mutation status (mutant or wild-type IDH), and 1p/19q co-deletion status (co-deletion or non-co-deletion). A nomogram model was established depending on the outcomes of the multivariate Cox regression, using the R package “rsm.” Continuous variables, the WHO grade of the LGG of the patient, and the ITGA2 expression level were contained in the nomogram model. Calibration curves were formed by means of the “calibrate” function of the “rms” package in TCGA cohort and were validated in the CGGA cohorts. Discrimination was evaluated by calculating Harrell’s C-index. To assess the clinical utility of the nomogram model, decision curve analysis (DCA) was used to compare the benefits of different factors.

### ssGSEA

Using an ssGSEA algorithm to quantify the enrichment of the 29 immune-associated signatures, downloaded from previous research (20, 21), and the R package “ GSVA,” we examined the infiltration of each immune cell type in the LGG tumor microenvironment (TME) (22). On the basis of the median expression of ITGA2, the samples were split into high- and low-expression groups for ssGSEA analysis in the TCGA cohort. The estimate algorithm was conducted to calculate tumor purity, estimate scores, immune scores, and stromal scores of LGGs using the “estimate” package (23). The relevant abundance of each infiltrating immune cell was calculated using the ssGSEA algorithm.

### Single-Cell RNA Sequencing Analysis

The distribution and abundance of ITGA2 in different cells, such as vascular cell, immune cell, neuron cell, astrocyte cell, neoplastic cell, oligodendrocyte cell, and oligodendrocyte precursor cell (OPC), were obtained by single-cell RNA analysis, implemented by R package “limma”, “Seurat”, “dplyr”, and “magrittr”.

### Tumor Immune Dysfunction and Exclusion

Tumor immune dysfunction and exclusion (TIDE) algorithm, based on the database of melanoma and non-small cell lung cancer, can predict the immune response of the patients in other cancer types (24). It mainly depends on two mechanisms: inducing the dysfunction of T cell in tumors with high cytotoxic T lymphocyte (CTL) abundance and preventing the infiltration of T cell in tumors with low CTL levels (25). TIDE algorithm was performed by the website http://tide.dfci.harvard.edu/. Depending on the expression data in the TCGA database, we estimate the immune response of patients with gliomas by utilizing the TIDE algorithm.

### Cell Culture

Bt142 mut/-, SW-1783, and SW-1088 human glioma cell lines were received from the American Type Culture Collection (ATCC). A normal human astrocyte (NHA) cell line was acquired from the Culture Collection of the Chinese Academy of Sciences (Shanghai, China). Leibovitz’s L-15 medium (ATCC), with 10% fetal bovine serum (Gibco) added, was used to culture SW-1783 and SW-1088 cell lines. Dulbecco’s modified Eagle’s medium/F12 (ATCC) medium was used to culture Bt142-mut and NHA cell lines. All cell lines were incubated in an incubator with 5% CO_2_ at 37°C.

### Western Blot Analysis and Quantitative Real-Time PCR

The use of human tissue was approved by the Ethics Committee of the Second Affiliated Hospital of Nanchang University. Cell and human tissue lysates were extracted with a radioimmunoprecipitation assay buffer (Solarbio, China) with a mixture of protease inhibitors. Western blot analysis was conducted using ITGA2 (1:1,000, 24552-1-AP, Proteintech, China) and glyceraldehyde-3-phosphate dehydrogenase (GAPDH) antibodies (1:5,000, 10494-1-AP, Proteintech, China). Briefly, the total cell lysates were isolated by 5–12% sodium dodecyl sulfate polyacrylamide gel electrophoresis and then transferred to polyvinylidene difluoride membranes (PVDF membranes, Millipore, MA, USA). The PVDF membranes were incubated with primary antibodies. Enhanced chemiluminescence was used to visualize specific proteins by GV6000M (GelView 6000pro; China). Total RNA was separated from cells using Simply P Total RNA Extraction Kit (Bioflux China), and then extracted RNA was reverse-transcribed to cDNA with Prime Script RTase (Ribobio, Guangzhou). FastStart Universal SYBR Green Master (Roche Diagnostics, Basel, Switzerland) was applied to conduct qRT-PCR. The nucleotide primer sequences are as follows: the forward primer for ITGA2 is 5′-TCCAGATGAGATTGATGAGACCAC-3′; the reverse primer for ITGA2 is 5′-AATCCATTCACGCAAACAGCA-3′; the forward primer for GAPDH is 5′-CTCACCGGATGCACCAATGTT-3′; and the reverse primer for GAPDH is 5′-CGCGTTGCTCACAATGTTCAT-3′.

### Immunofluorescence

SW-1088 glioma cell lines were fixed in methanol (Xilong Scientific, China), then permeabilized with 0.1% tween-20 (Solarbio, T8220, China), and blocked with 5% goat serum for 40 min. We stained SW-1088 cell using rabbit anti-ITGA2 (ITGA2, 1:200, 24552-1-AP, Proteintech, China). Then, we incubated it with AffiniPure Alpaca Anti-Rabbit IgG (H+L) antibody (1:200, min X Bov, Hu, Ms Sr Prot, Jackson ImmunoResearch, Japan), and the nucleus was stained by DAPI. The cells were measured by confocal laser scanning microscopy (Nikon, C2si/C2, Japan).

### Cell Proliferation Experiment

We designed the siRNA (http://biodev.extra.cea.fr/DSIR/DSIR.html) that target ITGA2 mRNA, transfected it into the SW1088 cell line using Lipofectamine™ 3000 Transfection Reagent (Thermo Fisher, L3000075, USA), and then incubated it in an incubator with 5% CO_2_ at 37°C. After transfection for 24 h, the SW1088 cell line was planted in a 96-well plate with a density of 2,000 cells/per well; 10 ul of CCK8 (Beyotime, C0037, China) was added to each well at 1, 2, and 3 days respectively, and the optical density value was measured at 450 nm after 2 h. The nucleotide siRNA sequence is 5′- GGAAGAGUCUACCUGUUUACU-3′ (sense strand).

### Cell Transwell Invasion Assays

Invasion analysis was performed using a Corning Transwell chamber and membrane with a pore size of 8 µM (Corning, 3524, USA). The chambers were coated with 500 ug/ml Matrigel (Corning, 356234 USA), spread with 30,000 cells on each chamber, and cultured with serum-free medium. Then, medium with 10% fetal bovine serum and 1% antibiotic/antimycotic was filled in the lower chamber of the transwell, and the 24-well plate was incubated in an incubator for 24 h. Next, we wiped the non-invasive cells on the surface of the chambers with a cotton swab, fixed the invasive cells with 4% paraformaldehyde (Solarbio, P1110, China) for 30 min, and dye them with Crystal Violet stain (Solarbio, G1075, China). Leica Microsystems D-35578 microscope was used to take the images of the invasion cells. The experiments were performed in duplicate.

### Statistical Analysis

The Kaplan–Meier method and two-sided log-rank test were employed to contrast the clinical outcomes between LGG subgroups with low and high ITGA2 expressions. The Kaplan–Meier method was adopted to execute survival analyses, which were compared with the log-rank test. The Student’s *t*-test or chi-square test was utilized to determine the differential expression of ITGA2 between different subgroups by clinical features, and these tests were also performed to probe the expression of various immune checkpoint genes in different subgroups (grouped by ITGA2 expression). A correlation analysis was implemented to confirm the relationship between ITGA2 expression and several immune checkpoint genes. On account of nonlinear dimensionality reduction, *t*-distributed stochastic neighbor embedding (tSNE) single-cell profiling was implemented by using the R package “Rtsne”. All statistical analyses were conducted by SPSS Statistics, version 25 (https://www.ibm.com/products/software; IBM, USA) and R programming, version 3.6.1 (https://www.r-project.org/). *P*-values less than.05 were considered as statistically significant.

## Results

### Clinical Relevance of ITGA2 in Patients With LGG

**Figure 1:**
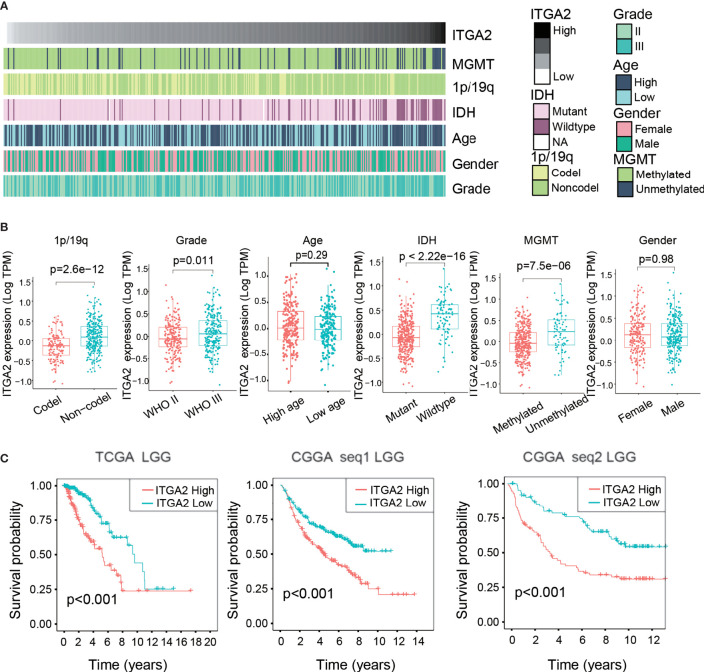
Clinical relevance of integrin alpha-2 (ITGA2) in patients with lower-grade glioma. **(A)** Relationship between ITGA2 expression profiles and clinical features of gliomas. **(B)** Variance analysis of ITGA2 expression in various clinical traits [gender, age, grade, *IDH*, 1p/19q, and O(6)-methylguanine-DNA methyltransferase] in The Cancer Genome Atlas (TCGA) cohort. **(C)** Prognostic analysis of different ITGA2 groups in a cohort from TCGA and two cohorts from the Chinese Glioma Genome Atlas, CGGAseq1 and CGGAseq2.

We screened out 407 patients with LGG in the TCGA cohort and 420 and 171 patients with LGG in the CGGAseq1 and CGGAseq2 cohorts, respectively. The related clinical information of selected patients with LGG are shown in **Supplementary Table S1**. A heat map displays the associations between ITGA2 expression and clinicopathological features in the TCGA dataset (**Figure 1A**). We then divided patients with LGG into subgroups of high and low ITGA2 expression in view of the median value of ITGA2 expression in the three independent cohorts and analyzed the relationship between ITGA2 expression and clinical information, including 1p/19q status, WHO grade, age, IDH status, MGMT status, and gender, in the three datasets. The results showed that the clinical characteristics had robust statistical differences, except for age and gender in the TCGA cohort (**Figure 1B**), and similar results were also observed in the CGGA cohorts (**Supplementary Figure S1**). These results suggested that the level of ITGA2 expression can influence the clinical characteristics of patients with LGG to some extent. Furthermore, we conducted a prognostic survival study on account of ITGA2 gene expression in the TCGA cohort and the CGGA cohorts (CGGAseq1 and CGGAseq2). The three independent cohorts displayed significant differences in survival outcomes (*P* <.001, **Figure 1C**), and the Kaplan–Meier survival curves suggested a consistent trend that patients with LGG had poor outcomes, usually accompanied with a high ITGA2 expression. These preliminarily data show that ITGA2 should be studied in more detail in patients with LGG.

### Verification of the Prognostic Value of ITGA2 in Three Cohorts

**Figure 2:**
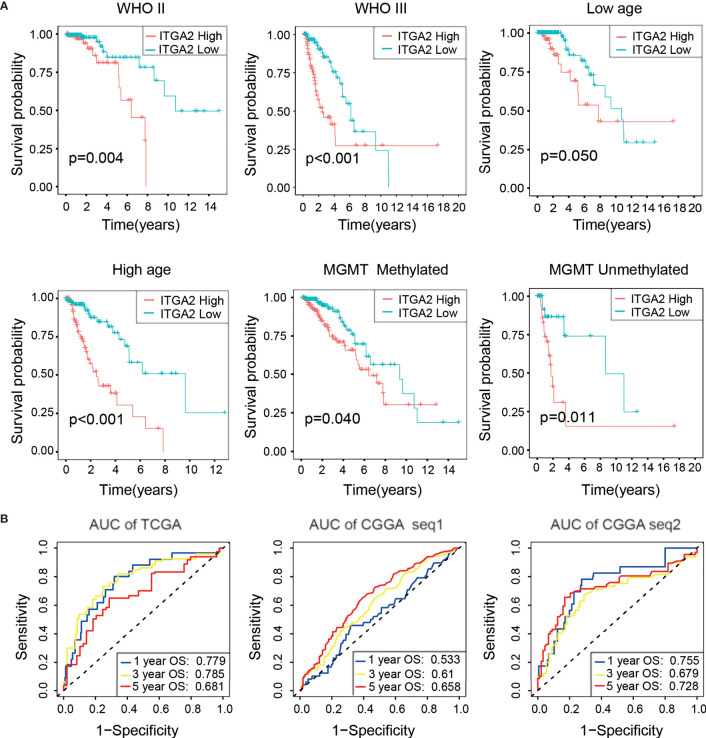
Verification of the prognostic value of integrin alpha-2 in three cohorts. **(A)** Kaplan–Meier overall survival curves of patients with lower-grade glioma grouped by integrin alpha-2 expression in The Cancer Genome Atlas (TCGA) dataset and stratified by World Health Organization grade, age, and O(6)-methylguanine-DNA methyltransferase status. **(B)** Time-dependent receiver operating characteristic curves for the prognostic model in a cohort from TCGA and two cohorts from the Chinese Glioma Genome Atlas, CGGAseq1 and CGGAseq2 (for predicting 1-, 3-, and 5-year overall survival).

We also performed a Kaplan–Meier analysis in each LGG subgroup, divided by clinical features, and the results indicated that the OS of the subgroup with low ITGA2 expression was always higher than the subgroup with high ITGA2 expression (**Figure 2A**). Similar results were obtained in the CGGAseq1 (*n* = 420) and CGGAseq2 (*n* = 171) cohorts (**Supplementary Figure S2**). Furthermore, we used the time-dependent ROC curves to analyze the accuracy of the prognostic model of patients with LGG in the TCGA, CGGAseq1, and CGGAseq2 datasets. As shown in **Figure 2**, the prognostic model of the TCGA cohort had outstanding accuracy with regard to 1-, 3-, and 5-year OS. The AUCs were 0.779, 0.785, and 0.681, respectively (**Figure 2B**). In addition, the AUCs of the prognostic model for 1-, 3-, and 5-year OS were 0.533, 0.610, and 0.658, respectively, in the CGGAseq1 dataset, and they were 0.755, 0.679, and 0.728, respectively, in the CGGAseq2 dataset (**Figure 2B**). These reliable results strongly displayed that ITGA2 could be a robust prognostic and predictive biomarker for patients with LGG.

### ITGA2 Is a Risk Factor in LGGs and *In Vitro* Experiments of ITGA2

**Figure 3:**
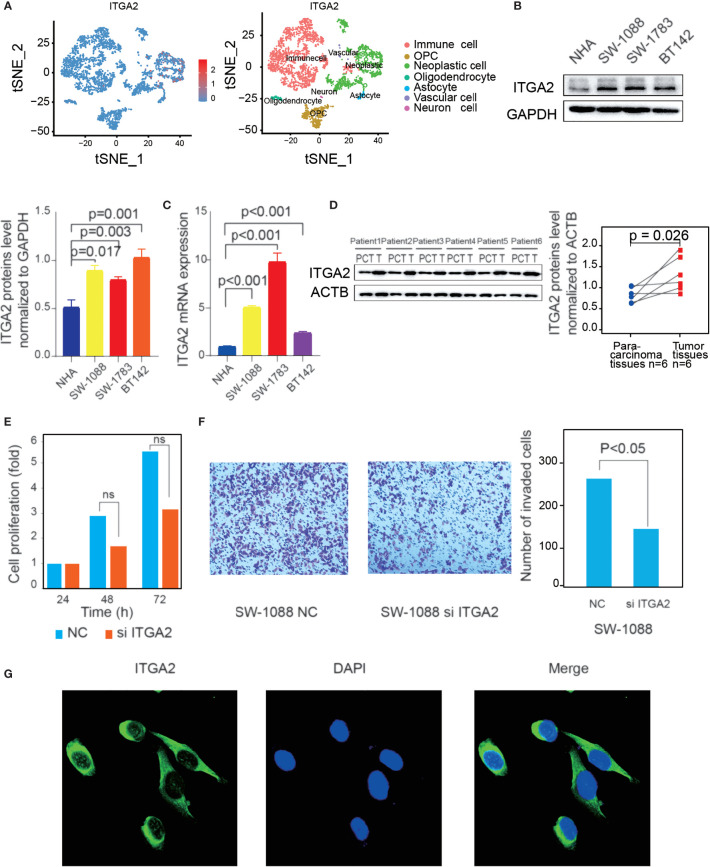
Verification of the high expression of integrin alpha-2 (ITGA2) in lower-grade gliomas (LGGs) and immunofluorescence of ITGA2. **(A)** Expression abundance of ITGA2 gene in different color-labeled cell types. **(B)** Western blot (WB) and **(C)** quantitative real-time polymerase chain reaction analysis of ITGA2 expression in LGG cell lines and normal glial cells (NHA). WB was repeated in three independent experiments. **(D)** Western blot analysis of ITGA2 expression in six paired LGG tissues and the match para-carcinoma tissue of the same patient. The ITGA2 protein expression levels were quantified by ImageJ software. WB was repeated in three independent experiments. **(E)** The effect of ITGA2 knockdown on SW1088 cell proliferation. Paired *t*-test was used to analyze. [ns (nonsense)] **(F)** The trans-well assays of SW1088 cell line under the knockdown of the ITGA2. The quantitative analysis of the transwell invasion assays performed by Image J Paired *t*-test was used to analyze. **(G)** SW-1088 cells were treated with the rabbit anti-ITGA2 (green) at 4°C overnight, and nucleus stained by DAPI. The protein plots were cut by the original bands.

Firstly, the tSNE single-cell profiling demonstrated that ITGA2 was more expressed in neoplastic cell than in other cell types, including immune cell, OPC, oligodendrocyte cell, astrocyte cell, vascular cell, and neuron cell (**Figure 3A**). Then, we detected the protein expression and mRNA expression in three LGG cell lines (SW1088, SW1783, and BT142) and in an NHA cell line (**Figures 3B, C****)**, which showed that ITGA2 expression was high in LGG cell lines compared with the NHA line. In addition, we collected LGG tissues and para-cancerous tissues from six patients in the Second Affiliated Hospital of Nanchang University. Compared with the matched para-cancerous tissue, ITGA2 proteins were overexpressed in LGG tissues, and the ITGA2 protein levels were also measured by ImageJ software (**Figure 3D**). We found that the knockdown of ITGA2 did not induce any statistically meaningful change in SW-1088 cell proliferation (**Figure 3E**). However, it reduced the invasion ability of SW-1088 cell. The number of invasion cells was qualified by Image J software (**Figure 3F**). The verification that ITGA2 was knocked down by siRNA at the protein level is shown in **Supplementary Figure S4**. Lastly, ITGA2 protein expressions were revealed by immunofluorescence staining. It was found that ITGA2 was mainly located in the cytoplasm and cell membrane (**Figure 3G**). These results demonstrated that ITGA2 was a risk factor in LGGs.

### Enrichment Analysis of ITGA2-Related Genes

**Figure 4:**
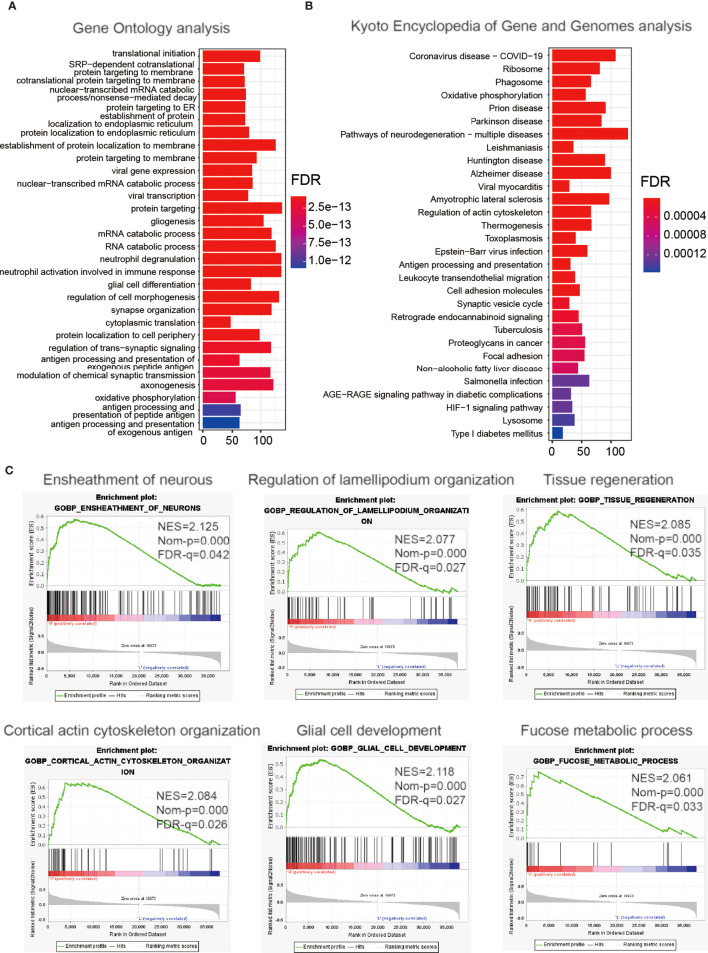
Enrichment analysis of integrin alpha-2-related genes. Functional analysis of 2,486 differentially expressed genes between the low- and high-expression groups. **(A)** Gene Ontology analysis of biological processes. **(B)** Kyoto Encyclopedia of Genes and Genomes pathway analysis. **(C)** Gene Set Enrichment Analysis.

To explore the impact of ITGA2 on the prognosis of LGGs in more detail, we made a differential expression analysis of all genes according to the ITGA2 expression, and we screened out the 2,486 DEGs [|log2 (fold change) | >1 and *P* <.05]. These DEGs were mainly used for GO-BP and KEGG analyses. The GO-BP study indicated that these DEGs enriched in co-translational protein biomarkers to membrane pathways, viral gene expression, and neutrophil activation involved in the immune response process, oxidative phosphorylation, and more (**Figure 4A**). The KEGG pathway research manifested that these DEGs were related to coronavirus disease of 2019, the severe acute respiratory syndrome coronavirus that has spread across the world (26) and associated with regulation of the actin cytoskeleton, proteoglycans in cancer, hypoxia-inducible factor 1 signaling pathway, and cell adhesion molecules (**Figure 4B**). The GSEA analysis indicated that tumor hallmarks were enriched in high ITGA2 expression subgroup, such as the ensheathment of neurons pathway, regulation of lamellipodium organization pathway, tissue regeneration pathway, cortical actin cytoskeleton organization pathway, glial cell development pathway, and fucose metabolic process pathway (**Figure 4C**). These data may offer some clues that could find the potential mechanisms of ITGA2 in LGG.

### ITGA2 as an Independent Prognostic Factor in Patients With LGG

We applied univariate and multivariate Cox regression analyses to estimate whether ITGA2 could be an independent prognostic factor in the training set and validating sets. Univariate Cox regression analysis indicated that ITGA2 was meaningfully correlated with clinical prognosis in patients with LGG in the TCGA cohort (hazard ratio, 2.2268; 95% CI, 1.7136–2.8936; *P* <.001; **Supplementary Table S2**). The multivariate Cox regression analysis then revealed that ITGA2 is an independent and powerful prognostic factor in TCGA datasets (hazard ratio, 2.0269; 95% CI, 1.1782–3.4871; *P* <.05; **Supplementary Table S2**). Similar results were obtained from the CGGA datasets (**Supplementary Table S2**). These results suggest that ITGA2 can be used as an independent clinical prognostic factor to predict OS for patients with LGG depending on the outcomes of the multivariate Cox regression analysis.

### Construction and Confirmation Clinical Nomogram in Patients With LGG

**Figure 5:**
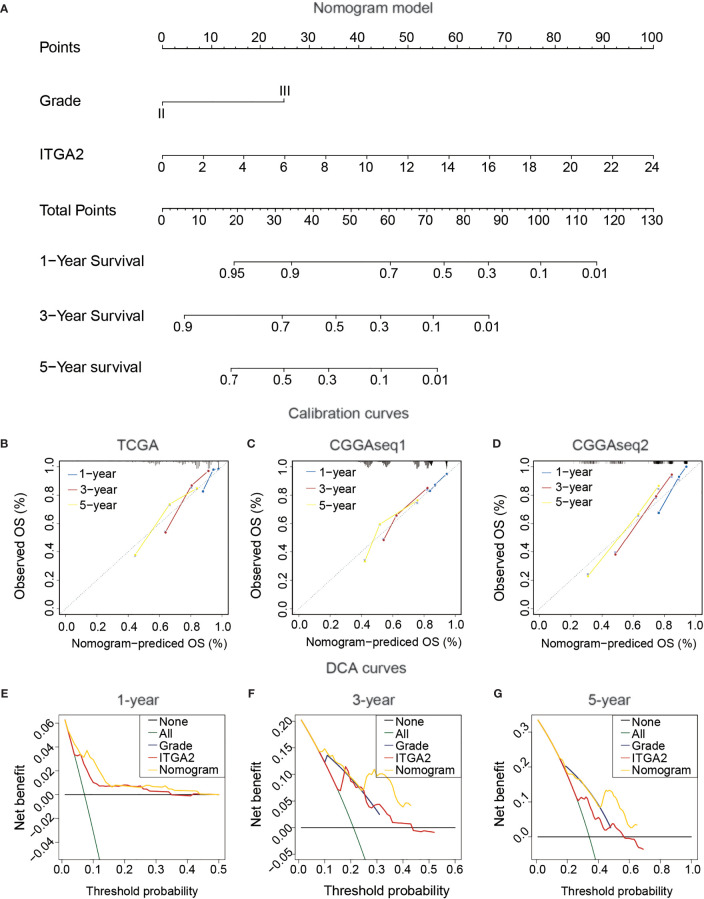
Construction and confirmation clinical nomogram in patients with lower-grade glioma. **(A)** The Cancer Genome Atlas (TCGA) nomogram model of clinical features, including *IDH* status, 1p19q status, grade, and integrin alpha-2 expression. **(B)** TCGA calibration plots of the nomogram. **(C, D)** The validation of the TCGA nomogram in Chinese Glioma Genome Atlas CGGAseq1 and CGGAseq2. **(E–G)** The validation of Decision Curves Analysis (DCA) in TCGA cohort. (for estimating 1, 3, and 5-year OS).

To produce a clinically usable and quantitative tool to predict the 1-, 3-, and 5-year OS of patients with LGG, we created a nomogram model using the ITGA2 expression and the WHO grade along with the outcomes of the multivariate Cox regression analysis (**Figure 5A**). The total points in the nomogram model forecast the 1-, 3-, and 5-year OS for patients with LGG. Then, we performed calibration curves to estimate the predictive ability of the nomogram model, and DCA was used to judge the net benefit for patients with LGG. The calibration curves of the nomogram model had a significant prediction accuracy for forecasting the 1-, 3-, and 5-year OS in the TCGA dataset (**Figure 5B**). The C-index reflects the predictive power of the nomogram between the TCGA dataset and the CGGA datasets; the results displayed a steady and powerful predictive power (**Figures 5C, D****)**. The C-index for the TCGA dataset was 0.804; for the CGGAseq1 dataset, 0.716; and for the CGGAseq2 dataset, 0.794. The DCA curves revealed that the clinical nomogram had robust accuracy for 1-, 3-, and 5-year OS compared with the other predictors in the TCGA cohort. In addition, the net benefit of the nomogram model was usually better than that of other predictors at various thresholds (**Figures 5E–G**). These powerful results prove that the nomogram model is accurate for use in patients with LGG, so this model may help identify high-risk patients in the future.

### The Correlation Analysis of Immune Infiltration

**Figure 6:**
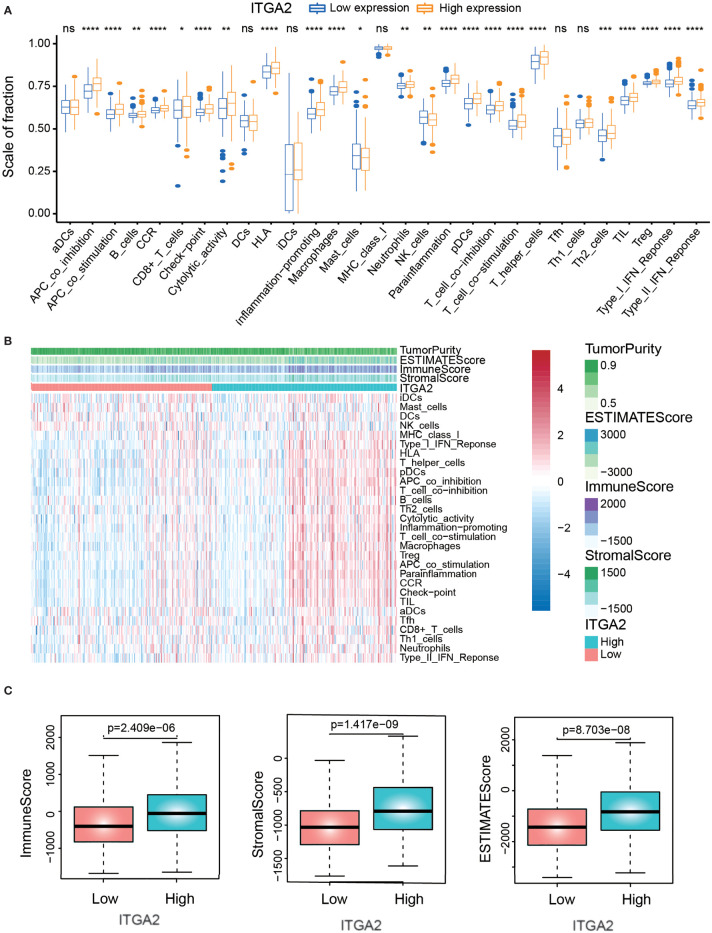
The correlation analysis of immune infiltration. **(A)** The differences of immune-related cells between low-expression integrin alpha-2 (ITGA2) and high-expression ITGA2 groups. **(B)** Heat map demonstrating the correlation between the expression of the ITGA2 genes and immune infiltration. **(C)** Correlation analysis between the expression of the ITGA2 gene and immune-related score (immune score, stromal score, and estimate score). *p < 0.05, **p < 0.01, ***p < 0.001, ****p < 0.0001. ns (nonsense)

To explore the relationship between immune infiltration and ITGA2 expression, we carried out the ssGSEA algorithm to quantify the enrichment of the 29 immune-associated signatures. Compared with the low-ITGA2-expression group, we found that the fraction of most immune-related signatures in the high-ITGA2-expression group was relatively higher (*P* < 0.05, **Figure 6A**). In addition, most of the immune features, such as presence of major histocompatibility class I, type I interferon response, and human leukocyte antigen status, were positively correlated with ITGA2 expression. The relevant abundance of the most of infiltrating immune cells increased as ITGA2 expression increased in the TCGA datasets (**Figure 6B**). Next, using the estimate algorithm to acquire the immune scores and stromal scores of patients with LGG, we implemented a differential analysis between the immune-related scores and ITGA2 expression. The results elucidated that ITGA2 was markedly correlated with immune infiltration: the immune-related scores were significantly different (*P* <.001) between the low- and high-ITGA2-expression subgroups (**Figure 6C**). These results indicated that ITGA2 is an immune gene that may have a certain impact on the prognosis of LGG.

### Analysis of the Correlation Between Immune Checkpoint and ITGA2 Expression Level

**Figure 7:**
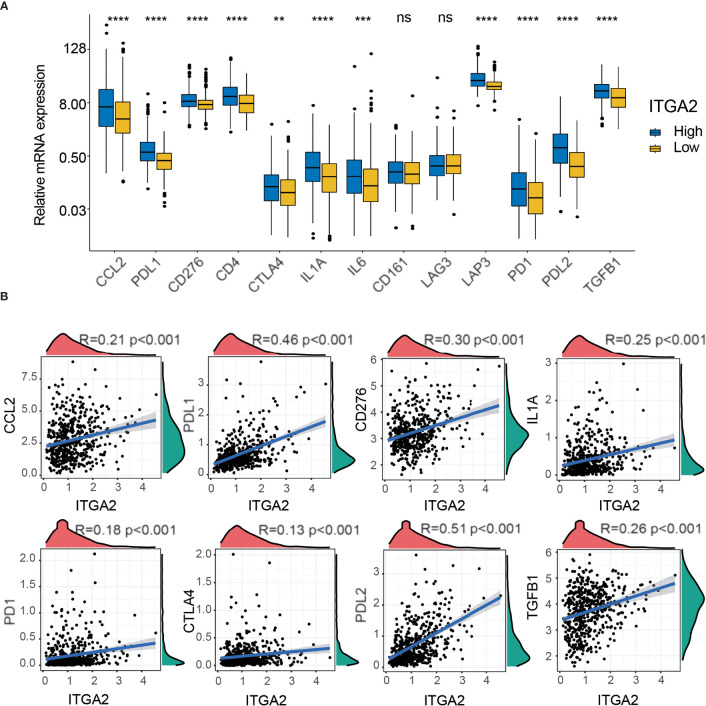
The correlation profiling of immune checkpoints. **(A)** Differential analysis of several immune checkpoint expression levels between high- and low-integrin alpha-2 (ITGA2) expression levels. **(B)** Correlation analysis between ITGA2 and eight selected immune checkpoint expression levels. **p < 0.01, ***p < 0.001, and ****p < 0.0001. ns (nonsense).

To detect the expression differences of various immune checkpoints between the subgroups with high and low ITGA2 expression in patients with LGG, we performed a differential study in the TCGA cohort. The result showed that most of the immune checkpoints, except the cluster of differentiation 163 and lymphocyte-activation-gene-3, differed significantly between the two subgroups (**Figure 7A**). To better understand the internal relationship between ITGA2 and known immune checkpoints, a correlation analysis was performed between ITGA2 and immune checkpoint expression in the TCGA cohort; we found that ITGA2 was prominently correlated with C-C motif ligand 2, programmed cell death 1 ligand 1 (PD-L1), cluster of differentiation 276, interleukin 1 A, programmed death 1 (PD1), cytotoxic lymphocyte antigen 4 (CTLA4), programmed cell death 1 ligand 2, and transforming growth factor beta 1 (**Figure 7B**). However, the correlation is insufficient, and the mechanism of ITGA2 at the immunosuppressive point still needs to be further explored.

### The Prediction of Clinical Immune Response Patients With Gliomas

**Figure 8:**
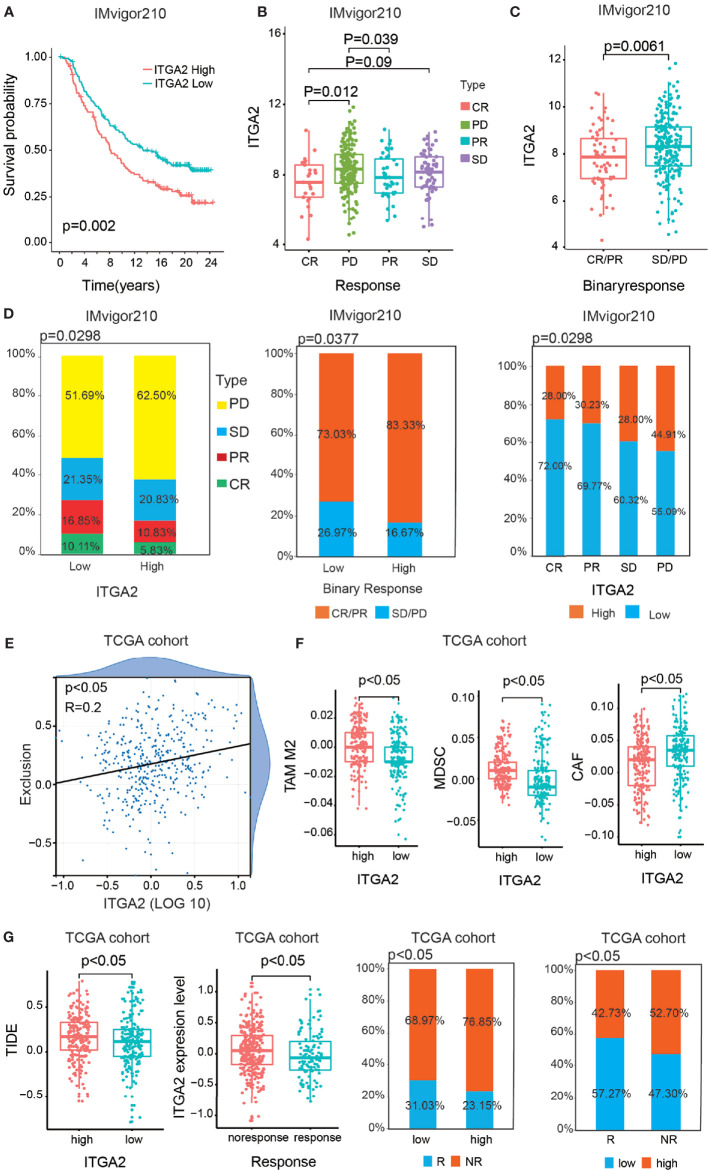
The statistics analysis of ITGA2 in the anti-PD-L1 (IMvigor210) cohort. **(A)** Kaplan–Meier overall survival (OS) curves of patients regarding the response of anti-PD-L1 (IMvigor210) cohort grouped by integrin alpha-2 (ITGA2) expression. **(B)** The variance analysis of ITGA2 in the distinct anti-PD-L1 clinical response groups. **(C)** The variance analysis of ITGA2 in the binary response. **(D)** The proportion analysis between ITGA2 subgroups and the distinct anti-PD-L1 clinical response groups. Chi-square test was used for data analysis. **(E)** The correlation analysis between ITGA2 expression level and the abundance of T cell exclusion. **(F)** The variance analysis of the TAM M2, MDSC, and CAF cells in the low- and high-ITGA2 groups. **(G)** The variance analysis of ITGA2 in the clinical responses and the variance analysis between ITGA2 subgroups and TIED score. The proportion analysis between clinical response groups and ITGA2 subgroups. Chi-square test was used for data analysis.

More independent cohorts ought to take for reverification the validated prognostic value of ITGA2. Therefore, we took the anti-PDL1 (IMvigor210) cohort for further validating the prognostic value of ITGA2. The anti-PD-L1 clinical response was grouped in progressive disease (PD), stable disease (SD), partial response (PR), and complete response (CR). Compared with the high-ITGA2 group, the prominent intensity of treatment and anti-PD-L1 immunotherapy response of the low-ITGA2 group were also validated (**Figures 8A–C**). The Kaplan–Meier survival curves suggested a consistent trend that patients with high ITGA2 expression level had poor outcomes (**Figure 8A**). We discovered that ITGA2 had significant differences between the CR and PD groups (*P* <.05, **Figure 8B**). ITGA2 had also meaningful differences between the CR/PR and SD/PD groups (*P* <.05, **Figure 8C**). In the anti-PD-L1 cohort, the proportions of CR, PR, SD, and PD were 10.11, 16.85, 21.35, and 51.69% in the low-ITGA2 group and 5.83, 10.83, 20.83, and 62.50% in the high-ITGA2 group, respectively. The proportions of CR/PR and SD/PD were 73.03 and 26.97% in the low-ITGA2 group and 83.33 and 16.67% in the high-ITGA2 group, respectively. The proportions of high ITGA2 expression in the CR, PR, SD, and PD groups were 28.00, 30.23, 28.00, and 44.91%, and the proportions of low ITGA2 expression in the CR, PR, SD, and PD groups were 72.00, 69.77, 30.32, and 55.09%, respectively **(****Figure 8D**). In addition, we also took the GSE78220 cohort and the GSE91061 cohort to predict the prognosis of ITGA2 in melanoma (**Supplementary Figure S3**). Due to the small sample size in the GSE78220 cohort, its results were not significant (**Supplementary Figure S3A**). In the GSE91061 cohort, the patients with high ITGA2 expression level have a poor outcome (**Supplementary Figure S3B**). Although the proportion analysis was not meaningless, the data were still different (**Supplementary Figure S3B**).

Based on the TCGA database, we also performed TIDE algorithm to reflect the different responses of patients to immunotherapy. In the previous study, the abundance of cancer-associated fibroblasts cells (CAFs), myeloid-derived suppressor cells (MDSCs), and the M2 subtype of tumor-associated macrophages (TAMs) can reflect the degree of T cell exclusion (24). Firstly, ITGA2 was positively interrelated to the T cell exclusion score (**Figure 8E**). Meanwhile, CAFs, MDSCs, and TAMs were also significantly different in the low- and high-ITGA2 groups (**Figure 8F**). Additionally, the results suggested that the higher TIDE score was accompanied by a negative clinical immune response, and the patients with low ITGA2 expression level had a more excellent clinical response than the high-ITGA2 group (**Figure 8G**). The proportions of response (R) and no-response (NR) were 31.03 and 68.97% in the low-ITGA2 group and 23.15 and 76.85% in the high-ITGA2 group, respectively. The proportions of low ITGA2 expression in the R and NR groups were 57.27 and 47.30%, and the proportions of high ITGA2 expression in the R and NR groups were 42.73 and 52.70%, respectively. These results indicate that LGG was realized as immune escape by preventing T cell infiltration, and the patients with low ITGA2 expression have a considerable immunotherapeutic response, which may be related to tumorigenesis and benefit by immunotherapies (27).

## Discussion

We studied 998 glioma samples from TCGA and CGGA cohorts to validate ITGA2 as a novel biomarker for LGG in both cohorts. The treatment of glioma demands on a multidisciplinary method, including neuroimaging, surgery, neuropathology, radiotherapy, chemotherapy, and supportive therapy (28). Clinical data from LGG patients are rare because of the low incidence rate, high mortality rate, and heterogeneity of multiple tumor subtypes (29). Therefore, traditional treatment is not always successful for patients with glioma, so it is urgent and pivotal to search for new molecular biomarkers to improve the prognostic outcome of patients with glioma. We undertook a series of systematic analyses to verify the prognostic value of ITGA2. First, we conducted a prognostic survival analysis and a relevant analysis of clinical and molecular characteristics for patients with LGG from three datasets. The patients with LGG with a higher ITGA2 expression had a worse prognostic clinical outcome compared with patients in the low-expression group. Next, ROC curves were used to judge the predictive power of the prognostic robustness in patients with LGG.

We then performed GO analysis, KEGG analysis, and GSEA. We found that the cancer-related indicators were more enriched in the LGG subgroup with high ITGA2 expression compared with the subgroup of low expression. Univariate and multivariate Cox regression analyses were used to prove the independent prognostic role of ITGA2 in LGG. In addition, we set up a nomogram model to forecast the prognosis outcome for patients with LGG depending on the outcomes of the multivariate Cox regression analysis; this model was validated by calibration curves and DCA. DCA is a novel statistical method that graphically describes the net benefit for patients at various thresholds. DCA was used to evaluate whether the nomogram model had utility in supporting clinical decisions and to determine which factors led to the best decisions. Therefore, DCA was an essential validation tool for clinical usefulness (30). Using the nomogram model, a neurosurgeon can better forecast the outcome of patients with LGG. To better comprehend the internal connections between immune infiltration and ITGA2 expression, we analyzed the levels of the immune score, stromal score, and estimate score between subgroups with low and high ITGA2 expression. The results suggest that ITGA2 is clearly related to immune cell infiltration. Finally, RT-qPCR and western blot were utilized to detect ITGA2 protein expression and mRNA expression in all three LGG cell lines (SW1088, SW1783, and Bt142) and in the NHA cell line; the results indicate that ITGA2 was expressed more in LGG.

The advantages of this study included the analysis of comprehensive clinical data from 999 LGG patients and IMvigor210 (*n* = 298) cohort. The construct of a nomogram model was validated in two external, independent LGG cohorts. The ITGA2-related signature has a strong and steady prognostic value, so it may be successful for clinical application. This study provides a powerful clue about predicting the prognosis and diagnosis of LGG patients. Nevertheless, the study has some limitations. More independent LGG cohorts ought to be taken for reverification of the validated prognostic value of ITGA2. For an expanded clinical application, the mechanism explaining the role that ITGA2 plays in affecting tumorigenesis and LGG development, specifically, must be determined. In addition, experimental studies, *in vitro* and *in vivo*, should be accomplished to explore the correlation between ITGA2-related signatures and outcome in patients with LGG.

It is now clear that TME contains not only cancer cells but also immune cells, stromal cells, endothelial cells, and cancer-related fibroblasts (31). After TME is formed, numerous immune cells can chemotax to there (32). Immunocytes may also produce a certain effect on tumors in a subtle way. TME has progressively been shown to indicate abnormal tissue function and to play a vital role in the subsequent progress of malignancies (33). In the recent years, immunotherapy has become a new research hotspot that can stimulate the actions of some immune cells in the TME to engender unknown effects on tumors. In addition, immune checkpoints block (ICB) treatment has become a new method to treat all kinds of cancers (34), especially the study of CTLA-4 and PD-1/PD-L1 molecules. In previous studies, some experts have already investigated a new mechanism by which ITGA2 plays a key role in regulating cancer immune response (35). Besides this, clinical studies have been carried out in lung cancer, prostate cancer, colorectal cancer, and melanoma treatment by ICB (36). It brings new challenges to the immunotherapy of gliomas. We found that ITGA2 may be interrelated to the immune response mechanism of cancer cells and was interrelated with TME. The proportion of immune cell infiltration in LGG was significantly correlated with clinical outcome. These details suggested that targeting ITGA2 may be an effective approach to improve the curative effect of cancer checkpoint immunotherapy. The results may offer some ideas for future research, concentrating on the mechanism of immune response processes.

It is reported that the abnormal expression of ITGA2 is involved in many cancers. One study indicates that a higher ITGA2 protein level was detected in breast cancers and that an increased expression of ITGA2 was positively bound up with increased metastatic ability in breast cancer (35). Another study showed that ITGA2 was highly expressed and may have had an extensive impact on colon cancer through interacting with transcription factors (37). In contrast to the expression in normal tissues, experts found that the mRNA expression of ITGA2 in gastric cancer was obviously increased. These results verified that targeting ITGA2 with antibodies not only inhibited cell migration but also induced an effect of apoptosis on gastric cancer cells (38). In contrast to normal glial cells, we also discovered that ITGA2 was notably upregulated in human LGG tumor cell lines. Growing evidence indicates that ITGA2 participates in the process of cancer pathogenesis and development. Therefore, we predict that ITGA2 may exert an influence on apoptosis, proliferation, and invasion in LGG. The existing standard treatments for LGG have limited effectiveness because of the aggressiveness of LGG and its resistance to chemotherapy. Thus, the discovery of a novel biomarker for patients with LGG is urgently needed. Comprehensive and systematic analyses revealed that ITGA2 may be a potential prognostic and predictive biomarker for LGG, which can bring new insights into targeted therapy. Moreover, ITGA2 was connected to immune infiltration; these data can provide researchers with new ideas about immunity in relation to the prognosis and diagnosis of patients with LGG. In conclusion, targeting ITGA2 may be a prospective immunotherapy to enhance the survival outcomes of patients with LGG.

## Conclusion

We suggested that ITGA2 can be a prognostic and predictive biomarker for patients with LGG. This study offered some ideas for future research, concentrating on the mechanism of glioma progression.

## Data Availability Statement

The datasets presented in this study can be found in online repositories. The names of the repository/repositories and accession number(s) can be found in the article/**Supplementary Material**.

## Ethics Statement

The studies involving human participants were reviewed and approved by the Ethics Committee of the Second Affiliated Hospital of Nanchang University. The patients/participants provided their written informed consent to participate in this study. Written informed consent was obtained from the individual(s) for the publication of any potentially identifiable images or data included in this article.

## Author Contributions

LL and ZT took the lead in writing the manuscript and figure production. LW, XZ, and KH supervised the work. JL contributed to data collection. KL, PW, ML, CG, and XL made important revisions to the paper. All authors contributed to the article and approved the submitted version.

## Funding

The current study was funded by the National Natural Science Foundation (grant nos. 81860448 and 82002660), the Natural Science Foundation of Jiangxi Province (grant no. 20192BAB205077), and the Jiangxi Province Department of Education Science and Technology Research Project, China (grant no. GJJ190018).

## Conflict of Interest

The authors declare that the research was conducted in the absence of any commercial or financial relationships that could be construed as a potential conflict of interest.

## Publisher’s Note

All claims expressed in this article are solely those of the authors and do not necessarily represent those of their affiliated organizations, or those of the publisher, the editors and the reviewers. Any product that may be evaluated in this article, or claim that may be made by its manufacturer, is not guaranteed or endorsed by the publisher.

## Supplementary Material

Supplementary Table 1 Clinical molecular information of patients with glioma involved in this study.

Supplementary Table 2 Univariate and multivariate Cox regression analyses based on clinical features (gender, age, grade, *IDH*, 1p/19q, and ITGA2) in three cohorts.

Supplementary Figure 1 Variance analysis of ITGA2 expression in various clinical traits. **(A, B)** Variance analysis of integrin alpha-2 (ITGA2) expression in various clinical traits (gender, age, grade, *IDH*, 1p/19q, and MGMT) in Chinese Glioma Genome Atlas CGGAseq1 and CGGAseq2 cohorts.

Supplementary Figure 2 The prognostic analysis of integrin alpha-2 in the Chinese Glioma Genome Atlas (CGGA) cohorts. **(A, B)** Kaplan–Meier overall survival curves of patients with lower-grade glioma grouped by integrin alpha-2 (ITGA2) expression in the CGGA CGGAseq1 and CGGAseq2 datasets, stratified by World Health Organization grade, age, and O(6)-methylguanine-DNA methyltransferase status.

Supplementary Figure 3 The statistics analysis of integrin alpha-2 in GSE78220 and GSE91061 cohorts. **(A)** Kaplan–Meier overall survival (OS) curves of patients regarding the response of the anti-PD1 (GSE78220) cohort grouped by ITGA2 expression and the proportion analysis between clinical responses and ITGA2 subgroups. **(B)** Kaplan–Meier OS curves of patients regarding the response of the anti-PD1 and anti-CTLA4 (GSE91061) cohort grouped by ITGA2 expression and the proportion analysis between clinical responses and ITGA2 subgroups.

Supplementary Figure 4 The verification of integrin alpha-2 knockdown at the protein level.
