# Supplementary material for: Integrin Alpha-2 as a Potential Prognostic and Predictive Biomarker for Patients With Lower-Grade Glioma
Source: Front Oncol. 2021 Oct 27;11:738651. doi: 10.3389/fonc.2021.738651 (PMC8578896; doi:10.3389/fonc.2021.738651)
Supplement: Supplementary file 1 [file DataSheet_1.docx]

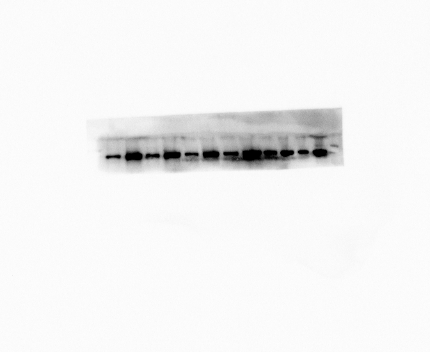

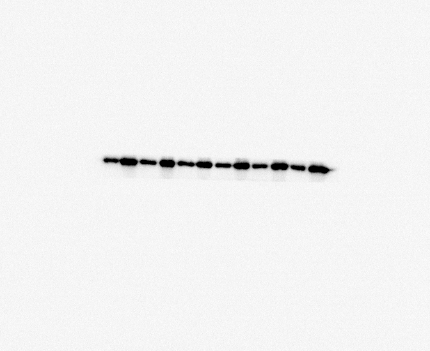

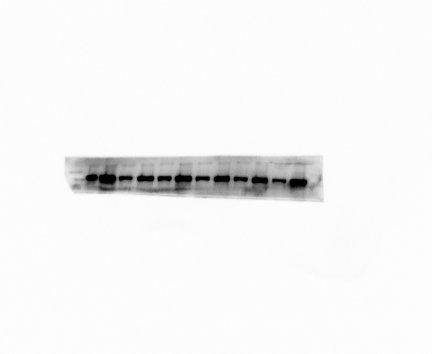


ITGA2 Plot Three times repeat


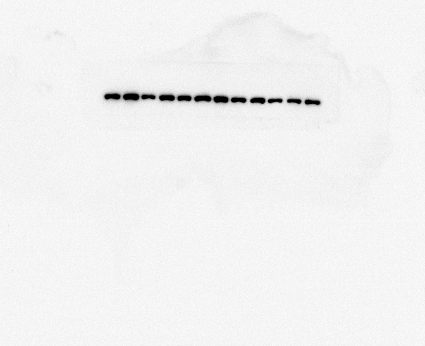

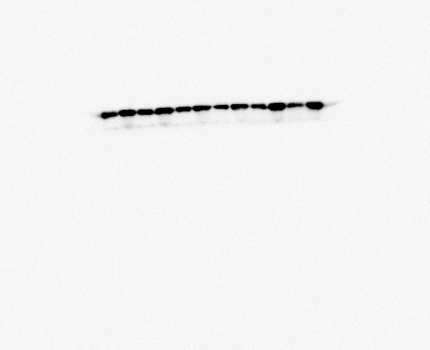

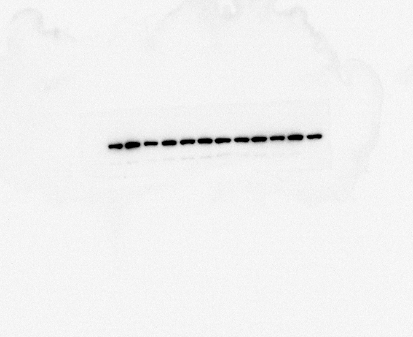


β-actin Plot Three times repeat


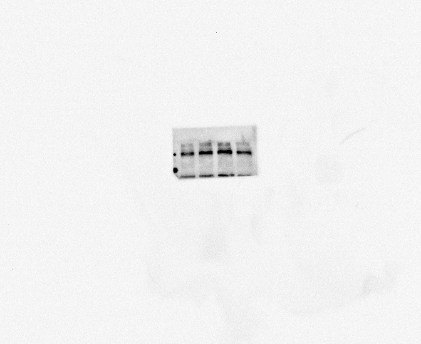

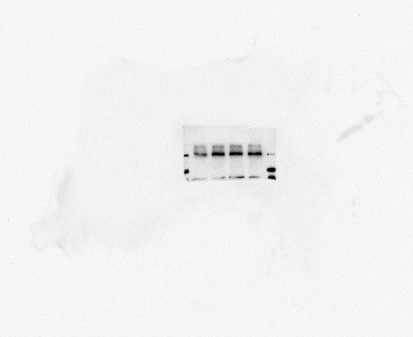

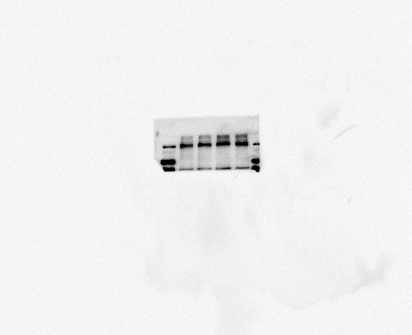


ITGA2 Plot Three times repeat


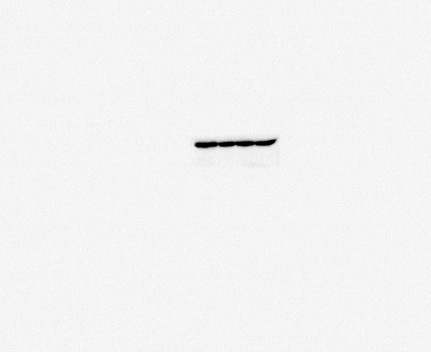

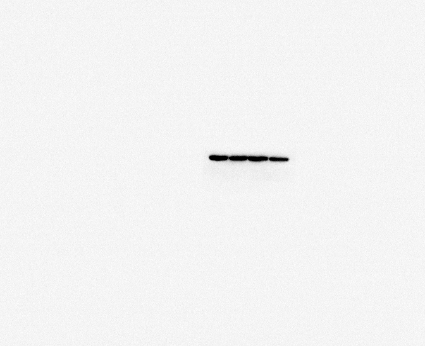

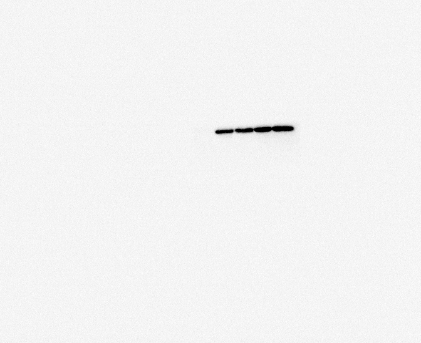


GAPDH Plot Three times repeat


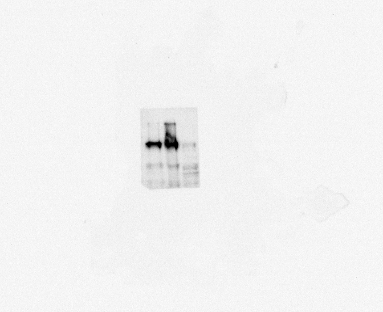

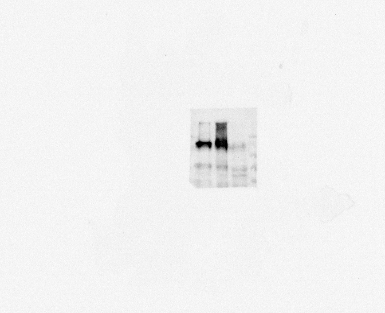

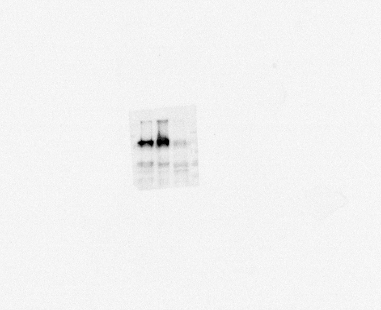


Si ITGA2 Plot Three times repeat


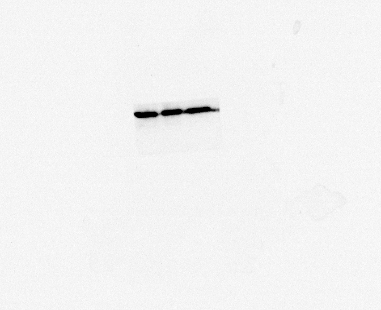

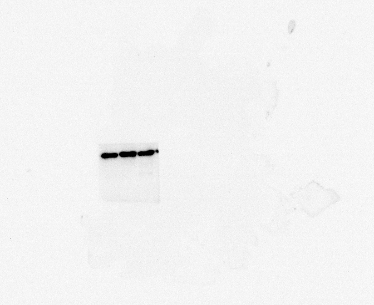

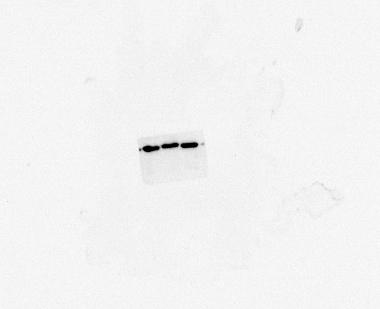


GAPDH Plot Three times repeat
