# Supplementary figures and images for: Integrin Alpha-2 as a Potential Prognostic and Predictive Biomarker for Patients With Lower-Grade Glioma
Source: Front Oncol. 2021 Oct 27;11:738651. doi: 10.3389/fonc.2021.738651 (PMC8578896; doi:10.3389/fonc.2021.738651)

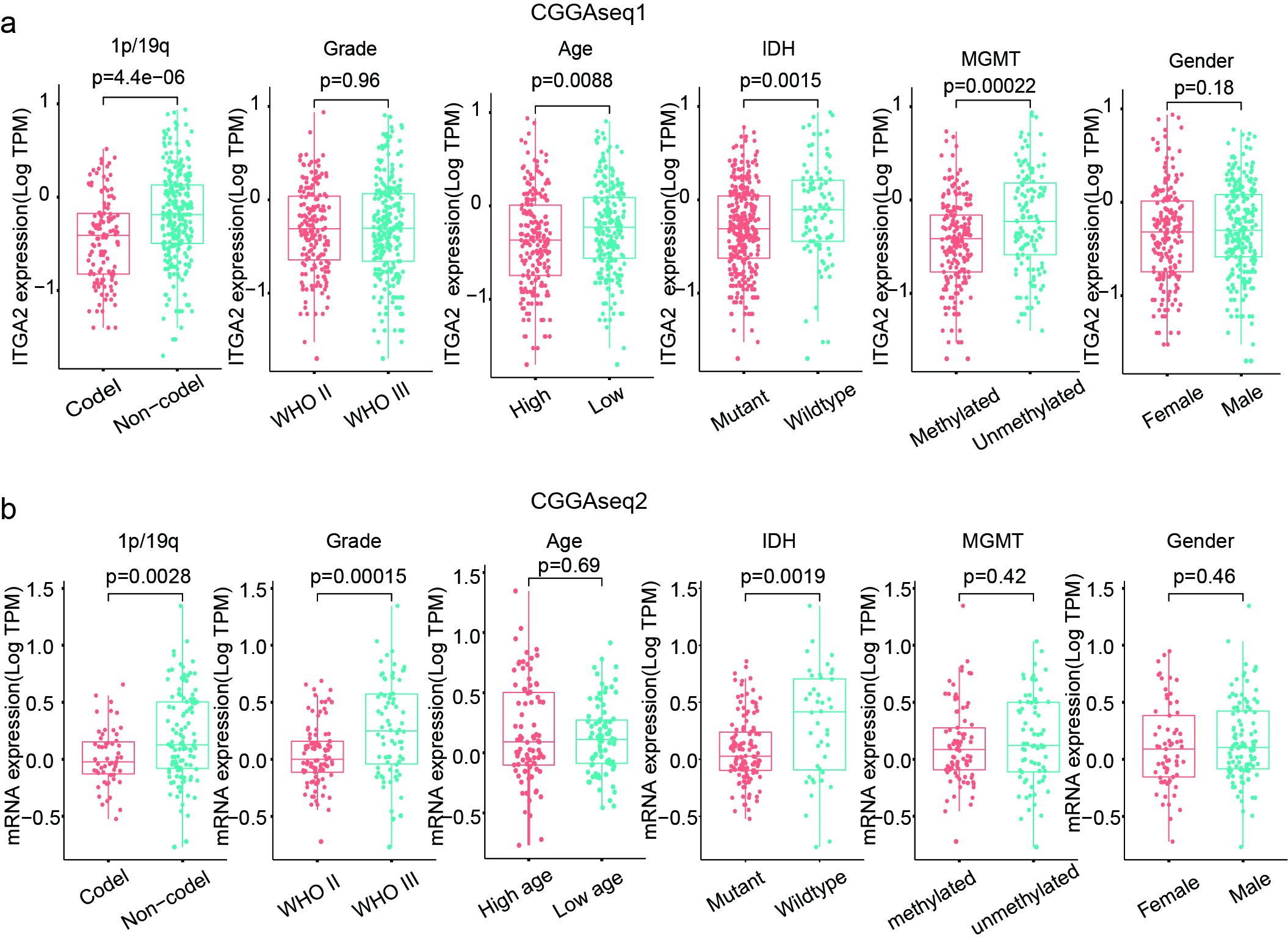

Supplement: Supplementary Figure 1 — Variance analysis of ITGA2 expression in various clinical traits. (A, B) Variance analysis of integrin alpha-2 (ITGA2) expression in various clinical traits (gender, age, grade, IDH, 1p/19q, and MGMT) in Chinese Glioma Genome Atlas CGGAseq1 and CGGAseq2 cohorts. [file Image_1.tif]

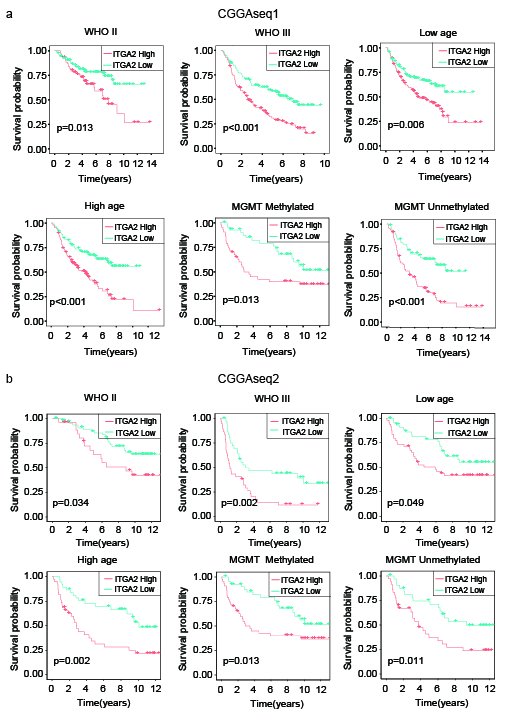

Supplement: Supplementary Figure 2 — The prognostic analysis of integrin alpha-2 in the Chinese Glioma Genome Atlas (CGGA) cohorts. (A, B) Kaplan–Meier overall survival curves of patients with lower-grade glioma grouped by integrin alpha-2 (ITGA2) expression in the CGGA CGGAseq1 and CGGAseq2 datasets, stratified by World Health Organization grade, age, and O(6)-methylguanine-DNA methyltransferase status. [file Image_2.tif]

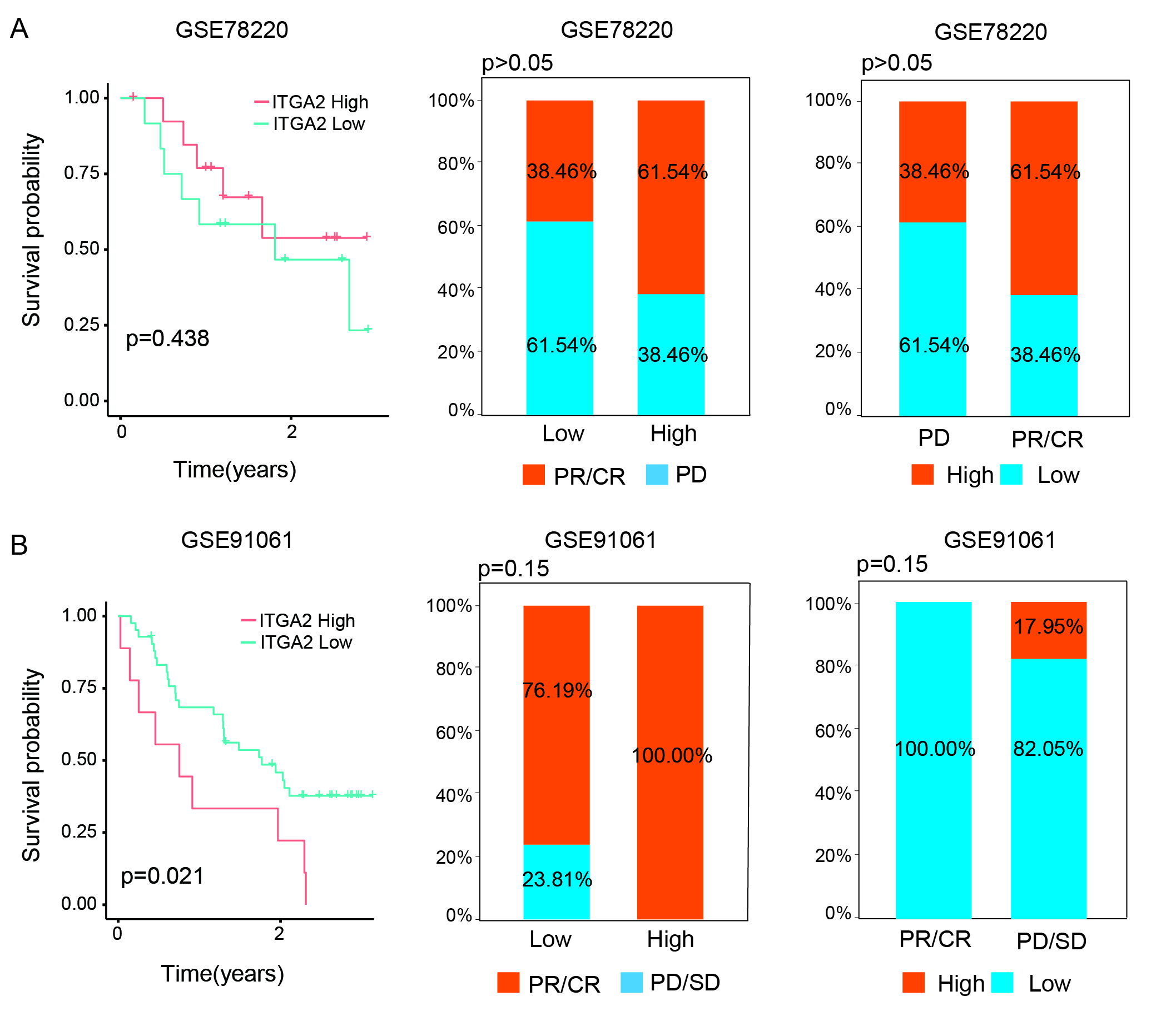

Supplement: Supplementary Figure 3 — The statistics analysis of integrin alpha-2 in GSE78220 and GSE91061 cohorts. (A) Kaplan–Meier overall survival (OS) curves of patients regarding the response of the anti-PD1 (GSE78220) cohort grouped by ITGA2 expression and the proportion analysis between clinical responses and ITGA2 subgroups. (B) Kaplan–Meier OS curves of patients regarding the response of the anti-PD1 and anti-CTLA4 (GSE91061) cohort grouped by ITGA2 expression and the proportion analysis between clinical responses and ITGA2 subgroups. [file Image_3.tif]

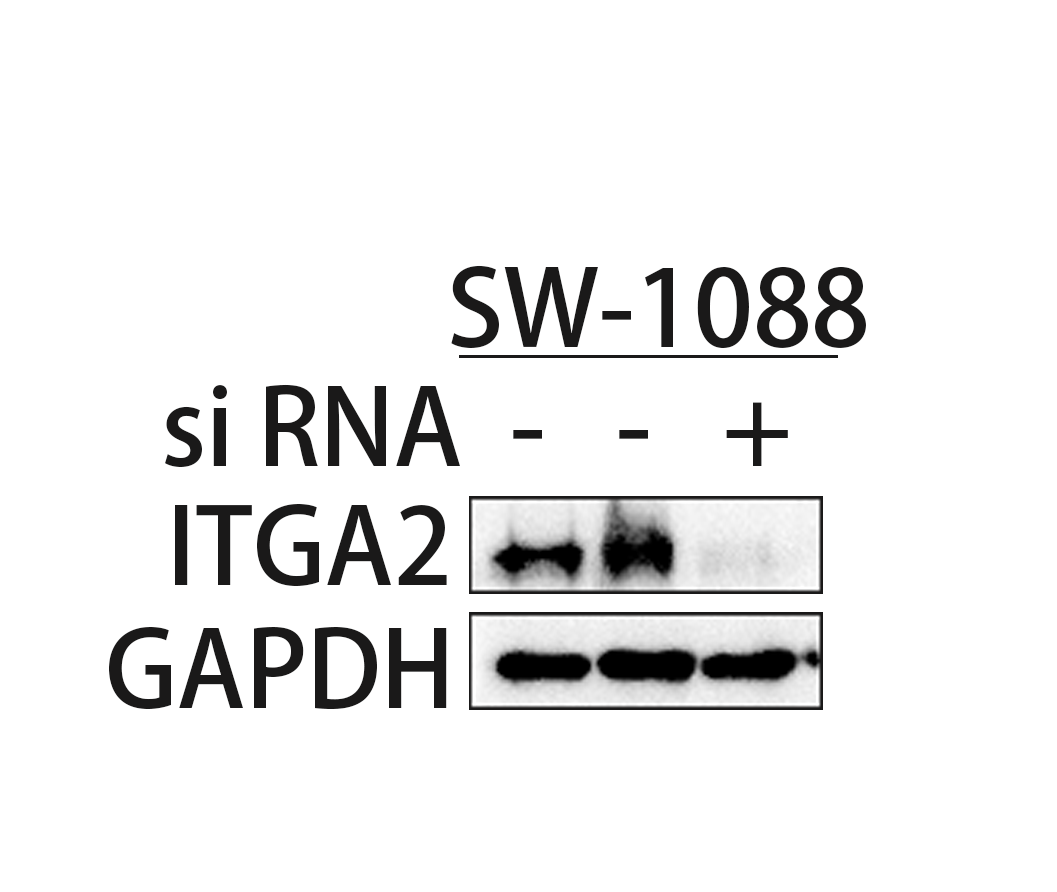

Supplement: Supplementary Figure 4 — The verification of integrin alpha-2 knockdown at the protein level. [file Image_4.tif]
